# Comparative Analysis of the Structural Weights of Fixed Prostheses of Zirconium Dioxide, Metal Ceramic, PMMA and 3DPP Printing Resin—Mechanical Implications

**DOI:** 10.3390/dj11110249

**Published:** 2023-10-26

**Authors:** Cristian Abad-Coronel, David Vélez Chimbo, Billy Lupú, Miguel Pacurucu, Marco V. Fárez, Jorge I. Fajardo

**Affiliations:** 1 Research Group on CAD/CAM Materials and Digital Dentistry, Faculty of Dentistry, University of Cuenca, Cuenca 10107, Ecuador; 2 Facultad de Odontología, Universidad de Cuenca, Cuenca 10107, Ecuador; davida.velez@ucuenca.edu.ec (D.V.C.); billy.lupuf@ucuenca.edu.ec (B.L.); miguel.pacurucu@ucuenca.edu.ec (M.P.); 3 New Materials and Transformation Processes Research Group GiMaT, Universidad Politécnica Salesiana, Cuenca 010105, Ecuadorjfajardo@ups.edu.ec (J.I.F.)

**Keywords:** structural weight, fixed prosthesis, zirconium dioxide, metal ceramic, PMMA, 3D printed, mechanical implications, 3D FEA, CAD/CAM materials

## Abstract

The aim of this study was to determine the mechanical implications of four-unit fixed dental prostheses (FDPs) made of (1) monolithic zirconium dioxide (ZR O^2^), (2) polymethylmethacrylate (PMMA), (3) metal ceramic (PFM) and (4) impression resin (3DPP). Methods: Four groups were studied with eight samples for each material (n: 32). Each structure was weighed, subjected to compressive tests and analyzed using 3D FEA. Results: PMMA presented the lowest structural weight (1.33 g), followed by 3DPP (1.98 g), ZR O^2^ (6.34 g) and PFM (6.44 g). In fracture tests, PMMA presented a compressive strength of 2104.73 N and a tension of 351.752 MPa; followed by PFM, with a strength of 1361.48 N and a tension of 227.521 MPa; ZR O^2^, with a strength of 1107.63 N and a tension of 185.098 MPa; and 3DPP, with a strength of 1000.88 N and a tension of 143.916 MPa. According to 3D FEA, 3DPP presented the lowest degree of deformation (0.001 mm), followed by PFM (0.011 mm), ZR O^2^ (0.168 mm) and PMMA (1.035 mm). Conclusions: The weights of the materials did not have a direct influence on the mean values obtained for strength, stress or strain. Since the performance was related to the tension and forces supported by the structures in critical zones, the importance of considering design factors is clear. In vitro and 3D FEA assays allowed us to simulate different scenarios for the mechanical properties of certain materials before evaluating them clinically. Thus, they can generate predictions that would allow for the design of a better research methodology in future clinical trials.

## 1. Introduction

Edentulism refers to the absence of teeth, either total or partial, and is considered a disability [1]. In a study by Polzer et al. carried out in 42 countries on the incidence of edentulism, prevalence rates between 1.3% and 78% were observed for people aged 74 years or older [2]. In clinical practice, there are a variety of options for the treatment of edentulism. One procedure with a high success rate is rehabilitation with fixed dental prostheses (FDPs), either on teeth or as implants [3].

An important topic is the fabrication material for FDPs, as it is advantageous to use lighter and stronger restorative materials. However, there is no information in the literature to support this statement [4]. Some of the characteristics of ceramics are their favorable esthetics [5], i.e., the fact that they imitate the natural shape and optical properties of teeth, as well as their chromatic stability, biocompatibility, high hardness, wear resistance and low thermal conductivity [6]. Another factor considered in the selection of the restorative material is the cost of the treatment [7]. Some of the advantages of FDPs are that they are comfortable for the patient, as they do not have to be removed; they are also adaptable and stable, and are biocompatible with the surrounding tissues [8]. Currently, due to advances in implantology and adhesive dentistry, there is a wide variety of FDPs available for replacing lost teeth [9] using different materials, such as zirconium dioxide (ZR O^2^), polymethylmethacrylate (PMMA) or impression resins for dental prostheses (3DPP) thanks to their mechanical, physical, biological and esthetic characteristics, in addition to conventionally used mixed materials such as porcelain fused to metal (PFM). Restorations can vary according to the size, design and dimensions of the preparations and specific indications for the use of the ceramic materials. With the application of digital flow through a computer-aided design and manufacturing (CAD/CAM) system, additive or subtractive restorations are currently fabricated with ceramic, plastic or hybrid materials with predetermined selection criteria for use in intraoral restorations due to their natural esthetics, high translucency and resistance to discoloration and wear [10].

### 1.1. Classification of Materials Used for the Study

**Figure 1:**
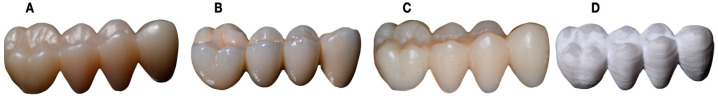
Four-unit FDP structures used for this investigation. (**A**) ZR O^2^ (KATANA, Zirconia STML), (**B**) PFM, (**C**) PMMA (Telio CAD) and (**D**) 3DPP (SprintRay ONX).

Four main types of restorative materials were analyzed in this study, and are described in Figure 1.

#### 1.1.1. Zirconia dioxide (ZR O^2^)

Zirconia dioxide has been used in dentistry due to its biocompatibility, adequate mechanical properties and better appearance with different indications, both for dental implants, such as abutments or crowns, and for fixed dentures [11]. Pure zirconium can present in three phases according to its chemical nature (monoclinic, tetragonal or cubic); the addition of dopants such as yttria keeps it in the metastable tetragonal phase. Thus, three generations of Yttria Tetragonal Zirconia Polycrystal (Y-TZP) have been used in restorative dentistry (3Y-TZP, 4Y-TZP, 5Y-TZP) [12]. In addition, less biofilm accumulates on the surface of ZR O^2^ [13]. It has a high flexural strength (900–1400 MPa) and high fracture toughness (5–10 MPa·m1/2) [14], which makes it possible to make crowns with reduced thickness (0.5 mm) [15]. This allows for a survival rate of up to 98% after five years compared to that achieved for metal–ceramic or fully ceramic FDPs, such as reinforced glass ceramics, glass-infiltrated alumina and glass-infiltrated zirconia alumina [16,17].

#### 1.1.2. Porcelain Fused to Metal (PFM)

A noble silver–palladium (Pd-Ag)-based alloy has been used for FDPs and has been considered the gold standard in restorative dentistry [18]. In addition, the alloys are partially or totally coated with feldspathic ceramics, which are in constant development [19]. Metals such as Cr-Co or Cr-Ni have also been used to replace noble and semi-noble metals.

The ceramic that covers the metal in an FDP is supported by a high-strength metal alloy core, lowering manufacturing costs [20]. To determine the functional performance, as well as the success and survival rates of PFM FDPs, a study was conducted that found survival rates of 98% after 5 years, 97% after 10 years and 85% after 15 years [21]. Some of the disadvantages of this restorative option include the dependence on shade selection and the need for manual application by the dental technician, chipping of the ceramic material, high weight, higher density, high thermal and electrical conductivity, long processing time and higher cost [22].

#### 1.1.3. Polymethylmethacrylate (PMMA)

Polymethylmethacrylate is commonly used for temporary FDPs, first introduced by Walter Wright in 1937 [23]. It consists of a layered polymer with satisfactory aesthetics, chemical stability and a low weight. In addition, it is corrosion resistant and hydrophobic. However, its mechanical properties are questioned due to fatigue caused by repeated masticatory forces, and by the propagation of microcracks in areas of stress concentration [24]. It is obtained using subtractive technology (milling) or additive technology (3D-printed resins) [25]. These technologies have come to replace conventional manufacturing methods with materials based on methacrylate resins with a liquid/powder content, or self-mixing composite resins [26]. Methacrylate resins initially have a self-curing chemical reaction, whereas composite-based materials can be found as self-curing, light-curing and dual-curing systems [27].

#### 1.1.4. D Printed Polymer (3DPP)

A digital workflow generates structures additively through the CAM process, obtaining a product via the accumulation of layers of material using 3D-printing technologies such as stereolithography (SLA), digital light processing (DLP), selective laser melting (SLM), selective laser sintering (SLS) and fused deposition modeling (FDM) [28]. The impression resin which is used requires minimal preparation, with slight touch-ups for finishing and termination; according to the manufacturer, it should be used for impressions of 50 micron resolution. Among its characteristics, it has a flexural strength of 147 MPa, a flexural modulus of 7986 MPa and an impact strength of 28 J/m^2^ [29].

### 1.2. Structural Weight of Materials

For the analysis of the weights of the elements used in the fabrication of FDPs designed for teeth or for implants, whether in ZR O^2^, PFM, PMMA or 3DPP, the strength-to-weight ratio of the materials used in their fabrication should be considered. This is because, in addition to compatibility, the weight influences the performance and functionality of the cemented FDPs. Therefore, it is necessary to consider the geometry of the implant/tooth or the FDP, and the material to be used to optimize its weight, mechanical resistance and effect on the structures adjacent to the teeth [30].

Mechanical tests are relevant in prosthodontics, and due to ethical considerations, in vivo tests are limited; the generation of virtual models by means of finite element analysis (3D FEA) overcomes these limitations and reduces the costs of the tests [31]. Through 3D FEA, it is possible to perform simulated structural calculations for FDPs, to which different conditions of mechanical and thermal loads and humidity conditions are applied, thus the effects of geometry and the weight of the material used on its resistance and useful life can be established [32].

Studies that demonstrate the effect of prosthesis weight from a mechanical point of view are limited. This research gap provides an interesting opportunity to acquire new knowledge. Among these studies, Tribst et al. (2020) evaluated the influence of denture weight on the microdeformation of bone tissue analyzed with different weights and numbers of implants. They reported that heavier prostheses, under the effect of the force of gravity, were related to greater stress being generated around the implants [33]. For their part, Skirbutis et al. (2017), in their review of the properties and use of polymeric materials such as poly ether ether ketone (PEEK), concluded that, compared to metals used in dentistry, PEEK is stable, biocompatible and light. It also has a reduced degree of discoloration; although, due to its grayish-brown color, its use is not indicated for restorations in the anterior sector [34].

Regarding metallic materials, Okubo et al. (2017) valued the use of titanium due to its low density, which offers opportunities for its use as a material for implant-supported restorative frameworks, improving mechanical properties [35].

Based on this background, the aim of this study was to determine and compare the structural weight, strength, tension and deformation of FDPs in different materials, such as ZR O^2^, PFM, PMMA and 3DPP. In this study, the null hypothesis was that there would be no significant differences in the structural weight, strength, stress or deformation of the FDPs of each studied material.

## 2. Materials and Methods

**Figure 2:**
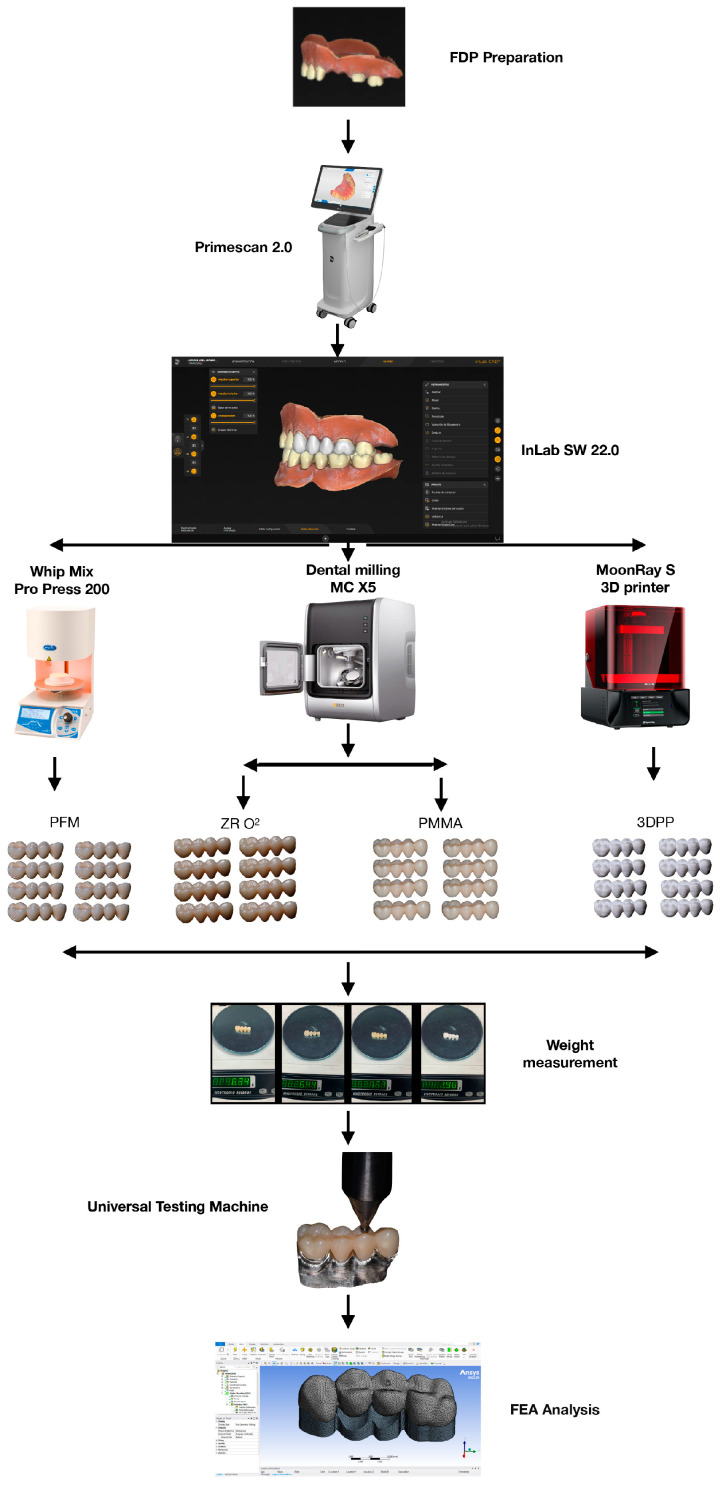
Outline of the methodology used in this study.

Four specific materials were used for this study: ZR O^2^ (KATANA, Zirconia STML, Kuraray Noritake Dental Inc., 300 Higashiyama, Miyoshi-cho Miyoshi, Aichi, Japan), PFM (metal; Wironia, BEGO, Bremen Gold Wilh. Herbst GmbH & Co., Detmold, Germany; VITA VM 13 ceramic, VITA Zahnfabrik, Bad Säckingen, Germany), PMMA (Telio CAD, Ivoclar Vivadent, Schaan, Liechtenstein) and an impression resin (3DPP) (SprintRay Pro 95, SprintRay, Los Angeles, CA, USA) (Figure 2)

A typodont was used, with preparations in pieces 2.3 and 2.6 of FDPs made of 4 units, and chamfer and parallelism of 6 degrees between its axial walls and rounded edges. Eight samples were made for each material (n: 32), while the PFM was obtained using the lost-wax technique and a manual stratified additive technique for ceramic on metal. For the other materials, a digital workflow was used. A digital impression of the typodont teeth was obtained with a stereo light scanner (PrimeScan 2.0, Dentsply-Sirona, New York, NY, USA) and subsequently transferred to software (InLAB SW 22.0, Dentsply-Sirona, New York, NY, USA) in which a design of the teeth was made. Once this process was completed, a 5-axis milling machine (MCX5, Dentsply Sirona, New York, NY, USA) and a 3D printer (SprintRay Pro 95, SprintRay, Los Angeles, CA, USA) were used to materialize the FDPs.

The laboratory analysis was performed once the restorations in the four types of materials to be studied had been been fabricated and adapted to the base model; each of these structures was weighed separately on a high-precision digital laboratory balance (Electric Balance LYC001, China).

### 2.1. Fracture Resistance Test

**Figure 3:**
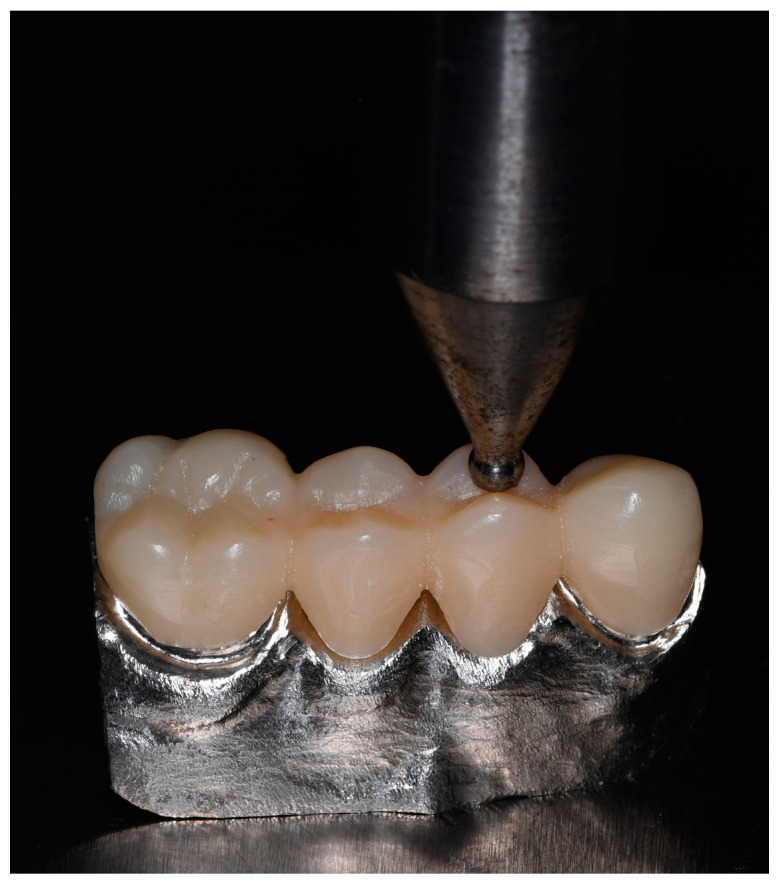
Application of compressive tests on FDPs’ structures, performed on a universal test machine.

To obtain and record the data necessary to perform the 3D simulations, the FDPs’ structures were subjected to fracture toughness testing by placing them on a beryllium-free nickel–chromium alloy metal die (Wirona, Bego, Goldschlägerei, Bremen, Germany), which was fabricated from the initial scan of the typodont; these frameworks were positioned on a universal testing machine (Shimadzu AGS-X series Universal Testing Machine, Shimadzu, Tokyo, Japan). Different units of compressive loads were applied following the direction parallel to the major axis of the occlusal face of the pontic in the FDP. The exact point was repeated in each of the structures and subjected to analysis using a hardened steel pin, with a radius of 3 mm, at a speed of 0.5 mm/min and an initial preload of 10 newtons (N), until failure of the frameworks occurred. At the beginning of the ache test, the equipment was calibrated to ensure equal conditions for each of the structures (Figure 3).

### 2.2. Simulation of Deformation of the Structures

After the fracture resistance test, a comparative study of the structural weights was carried out by means of analysis using 3D FEA software, (ANSYS Workbench R1, 2022, ANSYS Inc., Houston, TX, USA) in which solid geometric models were generated. They were then imported to the analysis software (ANSYS Workbench R1, 2022, ANSYS Inc., Houston, TX, USA) in standard format for the exchange of product data about where the tetrahedral elements formed in the analyzed mesh. Using this engineering software, simulations of the response to compressive loads under static conditions were performed. For this, the digital mesh of the FDP structures was generated, defining the study geometry in three volumes (die, metal base and polymer base). The mesh which was used contained 650,000 tetrahedral elements. A mesh quality of more than 85% was ensured in order to guarantee the results. Using this engineering software, simulations of the response to compressive loads under static conditions were performed using finite element Equation (1) for the model. The digital mesh of the FDP structures was generated, defining the study geometry in three volumes (die, metal base and polymer base).

(1) $$F=\left[k\right]U=\left[\begin{array}{ccc} k & -k & 0\\ -k & 2k & -k\\ 0 & -k & k \end{array}\right]\left\{\begin{array}{c} u_{i}\\ u_{j}\\ u_{k} \end{array}\right\}=\left\{\begin{array}{c} F_{i}\\ F_{j}\\ F_{k} \end{array}\right\}$$

where $u_{i},\;u_{j},\;u_{k}$ denote the nodal displacements, k is the stiffness of the crown material and $F_{i},\;F_{j},\;F_{k}$ represent the force components in each direction.

For each material, data on the configuration of the physical and mechanical properties of the materials were entered. The initial and boundary conditions were configured as shown in Table 1. In the 3D FEA, the stress analysis was performed using the Von Mises criterion, and the deformation was obtained.

## 3. Results

### 3.1. Experimental Results

**Figure 4:**
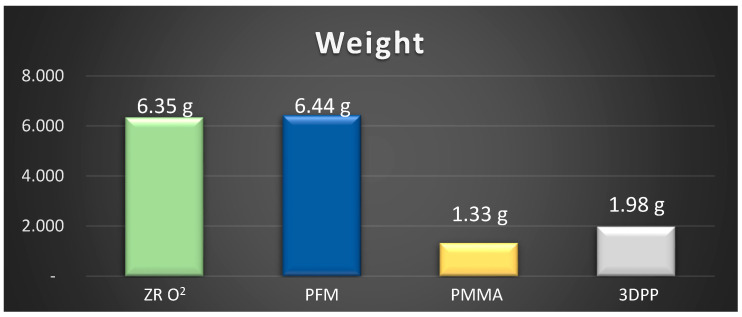
Comparative weight graph for each structure analyzed in this study.

Regarding the descriptive analysis related to the measurement of the weight of each of the structures, Figure 4 shows the observed results.

### 3.2. Fracture Resistance Test Results

The results obtained for each of the structures are presented in Table 2.

The descriptive analysis of the force (N) is summarized in Table 3. Although the PMMA and PFM materials showed much higher average forces, they also showed high coefficient of variation values, as did the Zr O^2^ and 3DPP materials.

### 3.3. 3D FEA Results

**Figure 5:**
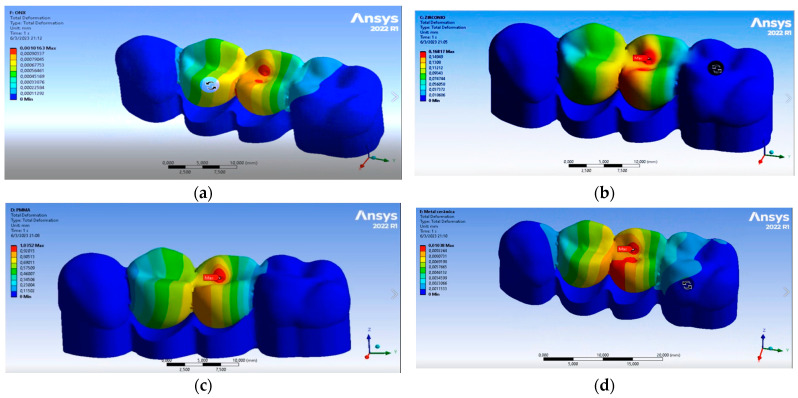
Deformation graphs for the structures during 3D FEA analysis; image (**a**) corresponds to 3DPP, image (**b**) corresponds to ZR O^2^, image (**c**) corresponds to PMMA and image (**d**) corresponds to PFM.

Regarding the 3D FEA simulation results, it was observed that 3DPP presented the lowest degree of deformation, in millimeters, compared to the other structures, followed by PFM, ZR O^2^ and, finally, PMMA. As for the stress supported by each of the structures, PMMA presented the highest value, followed by ZR O^2^, PFM and 3DPP (Figure 5) and the results are shown in Table 4.

Regarding the equivalent stress supported by the structure of the FDPs in cervical areas and connectors, the results, in MPa, are shown in Table 5.

The results of the descriptive stress analysis are shown in Table 6. The high coefficient of variation values suggest that the sample-to-sample measurements indicated high variability and low precision.

In terms of the descriptive data about deformation (%), the results are shown in Table 7.

### 3.4. Comparison of Experimental and 3D FEA Results

The data obtained from the comparison of the results of the in vitro experimental tests and the results of the 3D FEA simulation are shown in Table 8.

### 3.5. Inferential Statistical Test Results

The data were analyzed using the Statistical Package for the Social Sciences (SPSS) 27.0 (IBM Corp., Armonk, NY, USA).

The analysis was performed with a significance level of 5%. (a) The test statistics were adjusted for ties. (b) Multiple comparisons were not performed because the overall test showed no significant differences in the samples, the results are shown in Table 9.

Each row tests the null hypothesis that the distributions of sample 1 and sample 2 are equal. The significance level was 5%, the results are shown in Table 10.

## 4. Discussion

The materials used for fixed dental prostheses or implant-supported prostheses have increased considerably [36]. Years ago, the use of materials such as metal–ceramics was the gold standard; in recent years, the incursion of new technologies such as CAD/CAM has allowed the cost of the materials to be lowered. This leads to much faster and more efficient manufacturing of prostheses, increasing the quality of the treatments performed on each patient [37], thus encouraging the more frequent use of metal-free ceramics [38]. This topic of study has not been sufficiently investigated according to the current literature, this study being the first of its kind to analyze and document the mechanical properties of FDPs by means of 3D FEA simulations using four materials [39]. The values which were obtained allowed us to reject the null hypothesis that there would be no significant difference in the structural weights of fixed dental prostheses made of ZR O^2^, PFM, PMMA and 3DPP. In his studies, Tribst et al. mentioned that there is not enough information regarding the comparison of the mechanical effects on the maxillary bone when using a lighter prosthetic structure. Some authors state that zirconium dioxide structures are an evolution in the implant-supported metallic structures, taking into account the distribution of loads and forces that they could support, which are sometimes heavier than a cast metal structure [40]. For this reason, it was necessary to know whether the weight of the prosthesis could damage or benefit the bone tissue. According to Wolff’s law, depending on the magnitude of the tensions, this tissue can be deformed [41]. Thus, the choice of the material used for rehabilitation becomes important because the survival of the prosthesis will depend on this factor; the maximum forces and tensions supported will depend on the way in which they absorb the energy of the impact [42].

The null hypothesis was accepted because there were no differences between the force (N), strain (MPa) or deformation (%) of the prostheses for each material. No statistically significant differences between the forces (N) reported for each material were observed (H = 7.807; *p*-value ≥ 0.05), nor were significant differences between the strains (MPa) reported for each material observed (H = 7.807; *p*-value ≥ 0.05). Statistically significant differences between the strains (%) reported for each material were observed (H = 15.07; *p*-value < 0.05); thus, pairwise multiple comparison tests between materials were performed to determine groups that were homogeneous in terms of strain (%) values. In summary, PMMA had the highest mean values of force (N), stress (MPa) and deformation (%), as well as the lowest weight (1.33 g). PMMA was followed by PFM, with high values of force (N), strain (MPa) and weight (6.44 g), and with deformation higher than that of ZR O^2^ and lower than that of 3DPP. ZR O^2^ was third in terms of the force (N), strain (MPa) and weight (6.35 g) values, with the lowest strain value of all the materials. Additionally, 3DPP showed the lowest values of force (N) and strain (MPa), with a higher weight (1.98 g) than PMMA and a higher strain percentage than ZR O^2^. From these results, it was observed that the lightest materials, PMMA and 3DPP, presented the highest and lowest values of the study, respectively, a result that was similar for the heaviest materials, PFM and ZR O^2^. Thus, the fact that the weights of the materials did not seem to have a direct influence on the mean values obtained for strength, stress or strain does not seem to be correlated with the values of the mechanical properties analyzed. According to the results of this study, it was observed that the structure with the lowest percentage in mass, measured in grams, was PMMA, followed by 3DPP, ZR O^2^ and, finally, PFM; these materials being some of the most often used materials in prosthodontic rehabilitation [25]. This study shows that PMMA exhibits predominantly linear behavior compared to the other analyzed structures, exhibiting stress relaxation, plastic deformation and a modulus of elasticity dependent on the load and rate of load application. The plastic deformation of PMMA under different degrees of pressure could improve the obtained results; although, this would considerably increase the experimental stress, which is why it is necessary to implement compression tests [43]. The 3D FEA simulation used in this study confirmed that a heavier and stiffer material is not more resistant to forces or deformations, since it transfers more stresses to supporting structures, while the opposite occurs with more flexible and resilient materials [44].

As for the PFM ceramic coating, it had higher crystallinity and resistance than the ZR O^2^ ceramic coating, probably due to the incorporation of leucite to increase its thermal expansion coefficient [45]. It also presented better resistance to fracture loads, probably because its metallic substructure dispersed the stress of the ceramic coating [46]. In addition, it should be considered that the Young’s modulus of the metal was 150 GPa [47], while the Young’s modulus of ZR O^2^ was 210 GPa [48], demonstrating that the Young’s modulus is inversely proportional to the deformation [49].

The dynamic and impact forces in the mandible, applied against the jaws, are transferred in different ways for single, multiple and implant-supported prostheses. These forces depend on the material with which they are made: the most rigid materials, such as zirconium or metal–ceramic [50], generate a greater dynamic force load on the bone and adjacent structures compared to other materials used for prosthetic structures, such as carbon fiber, fiberglass or PEEK, which disperse and absorb dynamic forces and impact energy in better ways [51]. In relation to this, in the simulation using 3D FEA, 3DPP deformed the least; PFM showed less deformation than ZR O^2^; and PMMA was the material that deformed the most (Table 4). This was due to the manufacturing methods used for the printed materials, and caused by the addition of layers with chemical bonds between them. This process, together with the vertical orientation during construction, allowed the layers to be deposited perpendicularly to the direction of the application of the loads, improving their mechanical properties [52]. Tahayeri et al. mentioned that the lower the thickness of the printing layer of such 3D materials, the greater the number of layer-to-layer interfaces present in the structure. This allows them to polymerize in a better way, which improves the mechanical properties of the material [53].

**Figure 6:**
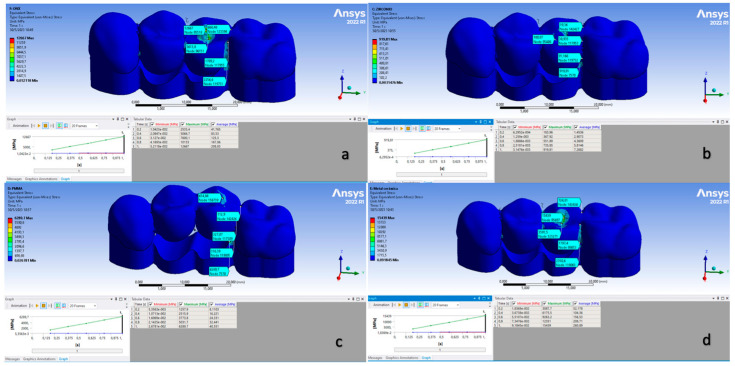
Graphics of 3D FEA material stress equivalent analysis: Image (**a**) corresponds to 3DPP, image (**b**) corresponds to ZR O^2^, image (**c**) corresponds to PMMA and image (**d**) corresponds to PFM.

According to the 3D FEA simulation conducted in our study, when comparing ZR O^2^ with PMMA, the former had lower deformation. This prevented stress dispersion in the ceramic structure, better supporting the compressive and deforming forces that would cause catastrophic failures in the FDPs. According to studies carried out by our research group, where milled restorations were compared with those obtained by 3D printing, the milled restorations showed a greater resistance to fracture (1663.57 N) in relation to those obtained from microhybrid resins using additive techniques (1437.74 N). These results are similar to those obtained in this study, demonstrating that restorations milled using CAM techniques present significant differences in terms of withstanding higher force loads and fracture resistance compared to restorations fabricated through 3D printing. This may be caused by the shrinkage that the material undergoes during processing and post-production [54]. The characteristics and conditions of the impact in the simulation were calculated in terms of velocity or acceleration and the displacement of an object, as well as the time and duration of the impact on the analyzed surfaces. In this study, the simulation of the application of compressive forces on each FDP structure lasted less than 1 millisecond; as indicated by previous works, this makes the software calculation more accurate [55]. Regarding the equivalent stress endured by each of the analyzed structures, from 0.25 milliseconds to after 1 s, it was observed that ZR O^2^ was the structure that suffered the least stress, followed by PMMA, 3DPP and, finally, PFM. Regarding the forces applied in MPa on the occlusal faces and connectors, the simulation showed greater stress in the connectors at the cervical level for the ZR O^2^ and PMMA structures, while the stresses in the occlusal area were greater for the 3DPP and PFM structures; this was due to the mechanical and physical characteristics of the materials, as can be seen in Figure 6 and Table 5. The specifications of several manufacturers regarding the minimum thickness that FDPs restorations are useful, because the behavior of a material is directly related to the stress that it can withstand at a certain thickness [56]. Even when using the suggested thicknesses, fractures or chips in the materials may occur; this problem can be predicted with the help of 3D FEA. This determines whether a material is able to withstand different masticatory loads before the fabrication of a dental prosthesis, and has been validated with real laboratory studies [57]. Connectors are fundamental in this type of restoration, so their size, in terms of both height and width, is crucial for their clinical success [58]. Chun et al. showed the maximum stresses accumulated in this area, recommending a minimum thickness of 0.8 mm for the connectors [59] to avoid bending and fracture, which would thus transmit less stress to the prosthesis and supporting structures [60]. Schmitter et al., in their prospective cohort study, evaluated the two-year clinical performance of extended tooth-supported zirconia, recommending 9 mm^2^ as the minimum size for connectors in the cross-sections of the FDPs [61]. They also concluded that triangular connectors presented greater resistance to bending from vertical forces, while circular connectors presented better results against oblique loads [62]. Therefore, the importance of the connector design as a factor in the predictability of FDPs is clear [63].

Regarding the thicknesses that ceramic crowns should be, Ozer et al. have suggested values of 1.3 mm in monolithic zirconium dioxide crowns, which have a similar resistance to metal–ceramic crowns; the flexural strength of zirconium increased as its thickness increased. Lower thicknesses were not recommended for the rehabilitation of the posterior sector since the crowns should be able to withstand forces of 500 N [64].

Regarding the comparison of the results obtained during the in vitro experimental tests using the 3D FEA simulation, it was observed that there were no significant differences in the data, as shown in Table 8, thus demonstrating the reliability and certainty of this type of simulation for more predictable treatments.

Among the limitations of the study is the fact that it was carried out in an in vitro environment; intraoral clinical application would be important in order to verify the forces applied to the structure and their influence on the structures studied. Another limitation is the application of static loads in only one direction, since clinical studies make reference to the fact that fracture loads that occur under static loading tend to present higher measurement values than those in humid environments and with dynamic loads [65]. A final important limitation of this study is related to the physical and mechanical properties of PFM since it is a material fused from several metallic and ceramic structures which caused complications when obtaining its characteristics in order to perform the simulation as it was not a monolithic structure.

## 5. Conclusions

Based on the results obtained in this study, the following conclusions are presented:

1. The structural weight of a material does not influence its greater resistance. A heavier structure would not be more resistant to stress or deformation, and may even transfer more stresses to the supporting structures; the opposite would occur with more flexible and resilient materials.

2. As shown in the results, and since the performance of a material is related to the stress and forces supported by the structures, especially in this area, the importance of considering design factors such as connectors and their shape and size is clear.

3. In vitro and 3D FEA assays allow for the simulation of different scenarios regarding the mechanical properties of certain materials before evaluating them clinically. Thus, they can generate predictions that may allow for the design of a better research methodology for future clinical trials.

## Figures and Tables

**Table 1:** Descriptive chart of the properties entered into the 3D FEA software for each material, obtained from the results of the stress tests.

Property	ZR O^2^	PFM	PMMA	3DPP
Density (kg m^−3^)	5900	3090	1200	2000
Young’s Modulus (GPa)	3.9	120	1.24	7.98
Poisson’s Ratio	0.24	0.24	0.38	0.28
Bulk Modulus (Pa)	2.50 × 10^9^	7.69 × 10^10^	1.72 × 10^9^	6.05 × 10^9^
Shear Modulus (Pa)	1.57 × 10^9^	4.83 × 10^10^	4.49 × 10^8^	3.12 × 10^9^

**Table 2:** Descriptive results for the vertical compressive forces, stresses and weights of each of the analyzed structures.

Material	Force (N)	Equivalent Stress (MPa)	Weight (g)
ZR O^2^	1107.63	18.5098	6.35
PFM	1361.48	22.7521	6.44
PMMA	2104.73	35.1752	1.33
3DPP	1000.88	16.726	1.98

**Table 3:** Force (N) descriptive data summary.

Material	Media	Standard Deviation	Coefficient of Variation	Min	Max
ZR O^2^	897.5477	379.4612	42.28%	477.390	1215.290
PFM	1728.8133	679.4625	39.30%	1123.010	2463.480
PMMA	1859.9027	620.6892	33.37%	635.786	2253.630
3DPP	858.0790	391.4379	45.62%	144.053	1343.810

**Table 4:** Descriptive results of stress and strain per structure in simulations performed using FEA software.

Material	Equivalent Stress (MPa)	Displacement (mm)
ZR O^2^	21.766	0.168
PFM	22.56	0.011
PMMA	37.374	1.035
3DPP	15.36	0.001

**Table 5:** Results of the equivalent of stress caused in the FDP’s structure obtained from the beginning of the 3D FEA simulation up to 1 s.

	0.25 ms	1 s
Equivalent Stress	Minimum MPa	Maximum MPa
ZR O^2^	183.96	919.81
PFM	3087.7	15.439
PMMA	1257.9	6289.7
3DPP	2533.4	12.667

**Table 6:** Descriptive summary of tension (MPa) for the 4 materials.

Material	Media	Standard Deviation	Coefficient of Variation	Min	Max
ZR O^2^	14.9999	6.3400	42.27%	7.9800	20.3090
PFM	28.8906	11.3546	39.30%	18.7669	41.1678
PMMA	31.0813	10.3725	33.37%	10.6248	37.6609
3DPP	14.3396	6.5414	45.62%	2.4073	22.4568

**Table 7:** Descriptive summary of strain (%).

Material	Media	Standard Deviation	Coefficient of Variation	Min	Max
Zr O^2^	2.0470	0.5582	27.27%	1.4475	2.5518
PFM	4.6828	1.9049	40.68%	2.8465	6.6496
PMMA	31.6752	9.5005	29.99%	13.6273	39.4554
3DPP	9.5779	2.9785	31.10%	4.0525	12.7738

**Table 8:** Descriptive results of the Von Mises stress equivalent (equivalent stress) comparison obtained in the in vitro tests and 3D FEA simulations.

	In Vitro	3D FEA
Materials	Equivalent Stress (MPa)	Equivalent Stress (MPa)
ZR O^2^	18.5098	21.766
PFM	22.7521	22.56
PMMA	35.1752	37.374
3DPP	16.726	15.36

**Table 9:** Summary of Kruskal–Wallis test results for independent samples.

Material	Test Statistic	*p*-Value
Flexural Strength (N)	7.807 ^ab^	0.05017
Equivalent Stress (MPa)	7.807 ^ab^	0.05017
Displacement (%)	15.07 ^ab^	0.00176

**Table 10:** Results of multiple pairwise comparisons for strain (%).

Outcome	Sample 1–Sample 2	Test Statistic	*p*-Value
Displacement (%)	ZR O^2^-PMMA	−13.500	0.000
	PFM-PMMA	−9.833	0.009
	3DPP-PMMA	6.333	0.040

## Data Availability

Data are contained within the article.
